# To What Are the Changes in the Color of the Blood Due

**Published:** 1874-11

**Authors:** E. M. Moore


ART. II.—*To What are the Changes in the Color of the Blood due.* By E. M. MOORE, JR., M. D.*

The fact that there exists a difference in the color of the blood of different animals, and of the same animal, was known for a long time, as would appear from the frequent mention of it, by writers of antiquity. Aristotle remarks upon the difference in the color of the blood of birds and fishes. Hippocrates noticed the difference of color in blood, as seen in the veins beneath the skin, and that which flowed from a wound. Galen also observed this and thought the blood underwent a change in color and believed this change took place in the liver. In 1553, Michael Servitus discovered that the blood changed its color in the lungs.

From the time of Priestley, there have been many theories held

*A Thesis recommended for publication, by the Faculty of the Buffalo Medical College.

concerning the color of the blood, in some, oxygen has played a prominent part, while in others it has been entirely excluded.

Lavoisier thought the difference in color of blood in veins and arteries was caused by a difference in the shape of the red globules in these vessels. That, in the arteries, they were bi-concave and therefore better able to reflect light, and that, in the veins, they lost the bi-concave shape, and consequently, the power to reflect light, to the same extent; hence the dark color. Mulder adopted this theory, and accounted for the bi-concave appearance of the red globules, by the formation of two oxides of protein in the lungs, which surrounded the globules, and contracting, caused them to assume the bi-concave shape, which they lost, together with their protein envelope, in passing through the systemic capillaries. Scherer considered the bright color of arterial blood due to the presence of white particles of chyle in the blood. Dr. Stevens,* in an article on the blood, says, the coloring matter of the blood is naturally dark, but is rendered scarlet, by the salts of serum. Also that bright arterial blood is rendered dark, by carbonic acid gas. That carbonic acid gas is formed in the capillaries, hence, the dark color, it is excreted in the lungs, hence, the bright color, which is not produced by oxygen, it having nothing to do with this change of color, in any way.

De Maack considered the color dark, and in no manner affected by oxygen or carbonic acid; but that the change was produced by the neutral salts, they producing two kinds of red, the first of an arterial hue, the latter of a venous tint.

Robinson† considered the change of color from arterial to venous due to carbonic acid.

Magnus considered it due to the same cause, and stated that the blood assumed the bright red color, when carbonic acid gas was removed. Engelhart was of the same opinion. Lehman and Meckel considered red color due to oxygen, and the dark color to carbonic acid, and this has been and is now the accepted theory.

It is the object of this thesis to sustain the theory, (advanced by Prof. W. H. Mason, of this University, during the winter of '72—'73,) that the color of arterial blood is red, from the presence

*Stevens, "On the Blood," London, 1832.

†Robinson, "On the Circulation of the blood," 1857.

of oxygen, in combination with the coloring matter of the red globules, and dark, in the veins, from the removal of it, to a certain extent, in the systemic capillaries.

The coloring matter of the blood is the hæmatine, which resides in the red globules. Its natural color is dark brown or black. It can be obtained in the form of crystals from blood. Its reactions without the body are similar to those, which occur in the blood, in the body during life, as far as its action with certain gases.

The gases of the blood, with the exception of nitrogen, which has since been demonstrated, as existing in that fluid, were first discovered by Magnus. They are oxygen, carbonic acid and nitrogen. But he found a disproportionately large amount of carbonic acid, and a correspondingly small amount of oxygen. This was due to an imperfection, in his method of obtaining them, which renders his statistics useless. According to Bernard, oxygen is found in the blood of the carotids, in the proportion of about 18 per cent., and in blood of the right side of the heart about 8 per cent. Ludwig gives the gas as existing in much larger quantity. In blood taken from the same places, arterial-oxygen 20 vols. in 100 vols., venous-oxygen 12 vols. in 100 vols. The amount of carbonic acid gas, according to Bernard is, in arterial blood, about 3 per cent., and in venous about 10 per cent. Of this gas also, Ludwig found a much larger per cent. In arterial 35 vols. per hundred—in venous 43 vols. per hundred. (This discrepancy in amount, was probably due to different conditions of the animals, from which the blood was taken.) Oxygen exists, in the blood, almost entirely in combination with the hæmato-globulins, but very little of it, being in solution in the plasma. While carbonic acid is in solution in the plasma and in combination with the alkaline carbonates, but very little of it being found in the red globules, and that produced by the process of nutrition, in the globule itself, and not having been absorbed from the plasma. If blood be deprived of its oxygen by means of a vacuum, it becomes of a dark color, although the carbonic acid has been removed at the same time. When any reducing agent be added to the blood, it is deprived of its oxygen, and assumed a dark color. If the oxygen be removed by hydrogen or nitrogen, the blood becomes dark. If the supply of oxygen be cut off from the blood, by tying the trachea, it rapidly becomes dark. If water be added to the blood, in proportion of one of the latter to three of the former, the globules are dissolved, the oxygen set free and the solution has a dark color. If venous blood be agitated with oxygen, it becomes red, now, if carbonic acid be passed through it for some time, and the blood be agitated with it, no change in color is produced, nor is there, even if the blood be allowed to stand in an atmosphere of carbonic acid, for twenty-four hours. If an animal be killed by oxygen, the color of both venous and arterial blood is bright red, although carbonic acid is still present. This occurs because oxygen is present in greater quantity than can be appropriated by the anatomical elements. The color of the blood, in veins coming from different parts of the body, varies, according to the amount of oxygen it contains. In veins leading from a gland in a state of activity, that is secreting, it is almost as red as the blood in the artery, leading to the organ. This is due to the fact, that the blood has parted, with but little of its oxygen. In veins, coming from muscles, in a state of activity, the blood is darker than it is in any other veins. This blood has parted with all of its oxygen. When carbonic oxide be added to the blood, the oxygen is driven off, but the color undergoes no change. Why this occurs is not known, but it is supposed, that carbonic oxide, by combining with the hæmato-globuline, acts towards it in the same manner as oxygen. Some state, that it is because the globules are still bi-concave, and thus reflect light the same, as when oxygen is present. However this may be, I am unable to state.

The conclusions to be deduced from the foregoing statements, are two:

*First*, that carbonic acid, in no way, affects the color of the blood. *Second*, that the presence or absence of oxygen causes the color of tarerial and venous blood.

*First.*—The coloring matter is naturally dark, and is contained in the red globules. Carbonic acid is not found in the red globules, except in a very small quantity, the result of the nutrition of the globule itself. It is present in arterial blood, at times, in so large an amount, as 35 vols. in 100 vols. If oxygenated blood be agitated with carbonic acid, no alteration in color is produced.

This proves almost conclusively, the first proposition, "that carbonic acid in no way, affected the color of the blood."

*Second.*—Oxygen exists in the globules, almost exclusively, in combination with the coloring matter of the blood. It reddens venous blood, and its extraction renders the blood dark again. In veins, from the blood of which but a small part of it has been abstracted, the blood retains its red color. When death is produced by oxygen, the color of the blood is everywhere red. This confirms the second proposition, "that the presence or absence of oxygen causes the color of arterial and venous blood.

